# A colorimetric immunoassay for the detection of human vascular endothelial growth factor 165 (VEGF_165_) based on anti-VEGF-iron oxide nanoparticle conjugation

**DOI:** 10.1007/s00604-024-06228-0

**Published:** 2024-02-14

**Authors:** Hülya Kuduğ Ceylan, Fatma Öztürk Kırbay, İdris Yazgan, Murat Elibol

**Affiliations:** 1 Department of Basic Pharmaceutical Sciences, Faculty of Pharmacy, Tokat Gaziosmanpaşa University, 60250 Tokat, Turkey; 2 Biochemistry Department, Faculty of Science, Ege University, Bornova, 35100 Izmir, Turkey; 3 Center for Biosensors and Material Science, Department of Biology, Faculty of Science and Art, Kastamonu University, 37100 Kastamonu, Turkey; 4 Bioengineering Department, Ege University, Bornova, 35100 Izmir, Turkey

**Keywords:** Human vascular endothelial growth factor, Iron oxide nanoparticle, Immunoassay, Indirect ELISA, Optical detection

## Abstract

Vascular endothelial growth factor (VEGF) is an indispensable element in many physiological processes, while alterations in its level in the circulating system are signs of pathology-associated diseases. Therefore, its precise and selective detection is critical for clinical applications to monitor the progression of the pathology. In this study, an optical immunoassay biosensor was developed as a model study for detecting recombinant VEGF_165_. The VEGF_165_ sample was purified from recombinant *Kluyveromyces lactis* GG799 yeast cells. Indirect ELISA was used during the detection, wherein iron oxide nanoparticles (FeNPs) were utilized to obtain optical signals. The FeNPs were synthesized in the presence of lactose p-amino benzoic acid (LpAB). VEGF_165_ antibody was conjugated to the LpAB-FeNPs through EDC/NHS chemistry to convert the iron oxide nanoparticles into VEGF_165_ specific probes. The specificity of the prepared system was tested in the presence of potential serum-based interferents (i.e., glucose, urea, insulin, C-reactive protein, and serum amyloid A), and validation studies were performed in a simulated serum sample. The proposed immunoassay showed a wide detection range (0.5 to 100 ng/mL) with a detection limit of 0.29 ng/mL. These results show that the developed assay could offer a sensitive, simple, specific, reliable, and high-throughput detection platform that can be used in the clinical diagnostics of VEGF.

**Graphical Abstract:**

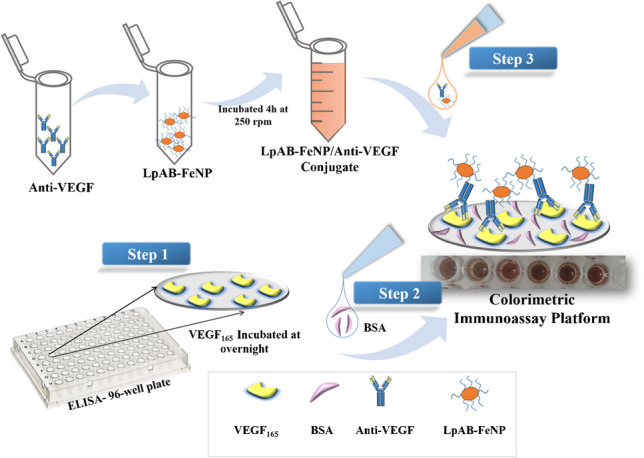

**Supplementary Information:**

The online version contains supplementary material available at 10.1007/s00604-024-06228-0.

## Introduction

Vascular endothelial growth factor (VEGF) is one of the most important cytokines that plays an essential role in vascular development and permeability [1]. VEGF and its receptors have been considered fundamental regulators for angiogenesis and vasculogenesis, which describe the processes of new capillary blood vessel formation [2]. VEGF triggers new blood vessel formation through the induction of vascular endothelial cells that can proliferate, migrate, and differentiate in healthy tissues [3, 4].

VEGF_165_ is the most dominant isoform of the human VEGF (also called VEGF-A) family and has the highest biological activity during angiogenesis [5]. Particularly, it participates in a variety of physiological angiogenic processes, including wound healing [6], female reproduction cycle [7], embryogenesis [8], neural development [9], bone formation [10], and hematopoiesis [11] in healthy adults. However, under certain circumstances, the overexpression of VEGF is well correlated with rheumatoid arthritis [12], Parkinson’s disease [13], psoriasis [14], and several ocular diseases [15, 16], while VEGF activity continues physiologically normal during vascular development. Besides, VEGF has also been widely described as a major tumor angiogenic factor in many tumor types [17, 18]. Particularly, VEGF participates in different stages of tumor, including development, progression, and metastasis, which can also orchestrate tumorigenic angiogenesis in ovarian [19], pancreatic cancer [20], lung (e.g., non-small cell lung cancer) [21], breast [22], oral [23], colorectal cancer [24], and liver cancers [25]. In this regard, elevated VEGF levels have been proposed as an essential biomarker in many types of cancers. In addition to cancer, such diseases as age-related macular degeneration in the eyes result in increased VEGF concentration in patients’ eyes. Therefore, the detection of the VEGF concentration in the patient’s retina is critical for the determination of the amount of anti-VEGF drug for intravitreal injections [26, 27].

Due to the critical importance of VEGF in biomedical research, early diagnosis, and medical applications, the detection and quantification of VEGF is strongly required to trace its levels in biological samples. Until now, a variety of approaches for the detection of VEGF have been reported, including enzyme-linked immunosorbent assay (ELISA) [28] (the standard assay format), fluorescence in situ hybridization (FISH) [29], immunohistochemistry (IHC) [30], and fluorescent spectrometry [31–33]. Even though each of these techniques has such superiorities, there is no available technique to overcome such drawbacks, including excessive time requirement, expensiveness, and requirements of complicated instrumentation. Among the analytical approaches in biosensor development, colorimetric techniques can overcome these shortcomings, which are commonly used for field analysis and point-of-care diagnosis for many applications. In the case of antibody-antigen-based reactions, antigen–antibody interaction causes alteration in the color intensity with favorable properties that can fulfill the desired requirements expected from a biosensor [32–34]. In recent years, colorimetry based on metal nanoparticles has garnered significant attention and has injected fresh vigor into traditional colorimetric methods, enabling precise and accurate analyzes [35]. Among the metal-based nanoparticles, iron oxide nanoparticles (FeNPs) provide colorimetric changes with many advantages as accuracy, simplicity, broad applicability, and high selectivity, which make them appealing for straightforward colorimetric detection. In addition, FeNPs are cost-effective compared to other metals and do not cause unstable and spontaneous aggregation when they are used as probes in detections [36, 37].

In this study, the synthesized FeNPs were conjugated with VEGF_165_ antibody for the colorimetric detection of VEGF_165_. The sensor gave a linear range between 0.5 and 100 ng/mL with 0.29 ng/mL as the limit of detection (LOD), which indicates that the developed immunoassay platform is highly promising for clinical applications. Furthermore, the sensing system gave excellent specificity for the potential interferents such as glucose, urea, insulin, C-reactive protein, and serum amyloid A in human serum.

## Materials and methods

### Reagents and strains

Recombinant *Kluyveromyces lactis* GG799 (New England Biolabs, Massachusetts, USA) was prepared by cloning the human VEGF_165_ cDNA (GenBank accession no. AF486837.1) in *Xho*I and *EcoR*I sites of the pKLAC2 vector under the control of the LAC4 promoter of the reported study [38]. The recombinant strain was cultured in YCB plates (3% 1 M Tris–HCl pH 7.4, 1.17% YCB, and 2% agar) containing 5 mM acetamide and maintained at – 80 °C as glycerol stock. In addition, the expression was performed using an unoptimized YPGal medium (1% yeast extract, 2% peptone, and 4% galactose). Ni–NTA agarose resin, 1000 kDa MWCO cellulose acetate dialysis membrane, and Pierce™ BCA Protein Assay Kit were purchased from Qiagen (Hilden, Germany), Spectrum Laboratories (California, USA), and Thermo Fisher Scientific (Waltham, MA, USA), respectively. N′-Ethylcarbodiimide hydrochloride (EDC) and N-hydroxysuccinimide (NHS) were purchased from Sigma-Aldrich (St. Louis, MO, USA) while C-reactive protein (CRP) (1 mg/mL) was obtained from Prospect. Serum amyloid A (SAA) (5.6 mg/mL) was purchased from Merck (Merck Millipore, Darmstadt, Germany). The washing buffer for ELISA was 0.1 M PBS (pH 7.2) containing 0.05% Tween 20 and 0.15 M NaCl. The coating buffer for ELISA was a 50 mM carbonate buffer, pH 9.6. ELISA maxisorp immunoplate (96 flat-bottom wells) was purchased from Nunc (Roskilde, Denmark). Artificial serum sample was prepared using KCl 0.335 g/L, MgCl_2_ 0.152 g/L, CaCl_2_ 0.505 g/L, NaCl 8.470 g/L, NH_2_CONH_2_ 0.150 g/L, D-glucose 0.450 g/L, and BSA 1.000 g/L as depicted in the literature [39].

### *Expression and purification of recombinant human VEGF*_*165*_

The inoculum was prepared by inoculating a single colony of recombinant *K. lactis* GG799 strain in 25 mL YPGal medium and incubated at 30 °C at 250 rpm for 72 h. The culture was inoculated with a ratio of 1 to 100 in a 2 L Erlenmeyer shake flask including 300 mL YPGal, which was then followed by, and then, the expression of VEGF_165_ was performed. After 48 h incubation, the culture suspension was centrifuged at 5000 rpm for 5 min at room temperature. The supernatant containing the secreted recombinant VEGF_165_ was utilized to purify the protein to use a standard protein for ELISA studies. During the purification process, the supernatant was precipitated with 60% ammonium sulfate solution in an ice bath for 60 min and then centrifuged at 12,000 rpm for 20 min. One hundred millimolars of phosphate buffer (pH 7.4) was used to dissolve the obtained protein pellet. After adding Ni–NTA agarose resin to the polycarbonate column to perform affinity-based purification chromatography, the column was washed with a 100 mM phosphate buffer (pH 7.4). The protein solution was applied to the column, and the target protein bound to the column was obtained through fractionations using the same phosphate buffer containing 300 mM imidazole. The recombinant VEGF_165_ elution solution was dialyzed using 1000 kDa MWCO cellulose acetate dialysis membrane against HEPES buffer (20 mM pH 7.4) overnight + 4 °C on a magnetic stirrer. The concentration of VEGF_165_ was calculated using the “Pierce™ BCA Protein Assay Kit.”

### Synthesis and characterization of LpAB-FeNP

Synthesis of iron oxide nanoparticles was performed using a modified version of the reported studies [40, 41]. Briefly, 1 M citric acid was mixed with 100 mM of FeCl_3_, 50 mM of FeSO_4_, and 10 mM lactose p-aminobenzoic acid (the sugar ligand was provided by Dr. İdris Yazgan of Kastamonu University, Turkey). The mixture was then vortexed at 1200 rpm, and 1 M of NaOH was added to the mixture while vortexing at 600 rpm. The addition of NaOH resulted in the formation of heat, which played a vital role during the iron oxide nanoparticle formation. The colloidal nanoparticles were then transferred to a new sterile 15-mL centrifuge tube. The goal of the addition of the sugar ligand (the synthesis approach can be found elsewhere [42]) was to enhance the stability of the synthesized iron oxide nanoparticles [43] and introduce an amine group. UV–vis spectroscopy, high-resolution transmission electron microscopy-selected area diffraction (HRTEM-SAED), scanning electron microscopy (SEM), and X-ray photoelectron spectroscopy (XPS) were used to characterize LpAB-FeNPs.

### Conjugation of anti-VEGF with LpAB-FeNP

Anti-VEGF conjugation to LpAB-FeNP was performed using the EDC/NHS chemistry method [44]. Anti-VEGF was activated using 50.0 mM EDC and 12.5 mM NHS and then introduced to LpAB-FeNP solution. Anti-VEGF:EDC:NHS (1:2:2; v:v:v) was mixed in PBS and incubated at 250 rpm in an orbital shaker for 15 min. After that, the LpAB-FeNP/anti-VEGF conjugate was washed three times with PBS buffer using 50-kDa centrifugal filters at 4000 rpm. Dynamic light scattering technique (DLS, Malvern Zetasizer Nano ZS model) was used to measure the size distributions and zeta potential of LpAB-FeNP and LpAB-FeNP/anti-VEGF.

### Construction of the immunoassay platform

First, the purified recombinant VEGF_165_ (1 mg/mL) was diluted to 10 different concentrations (0.5, 1.0, 5.0, 10, 25, 50,100, and 250 ng/mL) with coating buffer and added onto the ELISA plate. This plate was then incubated overnight at 4 °C. After that, unbound VEGF_165_ was removed from the plate by cleaning it with a washing buffer. Next, 2 mg/mL BSA was added to each well and incubated for 1 h at room temperature as a blocking step. The conjugate of LpAB-FeNP/anti-VEGF was introduced to the plate and incubated for 1 h. After the incubation, a washing buffer was used to remove the unreacted components. The UV–Vis absorbance of this system was measured, and the absorbance varied according to the VEGF_165_ concentration. The proposed immunoassay platform was similar to a previous study [45].

## Results and discussion

### Characterization of LpAB-FeNP and LpAB-FeNP/anti-VEGF

**Fig. 1:**
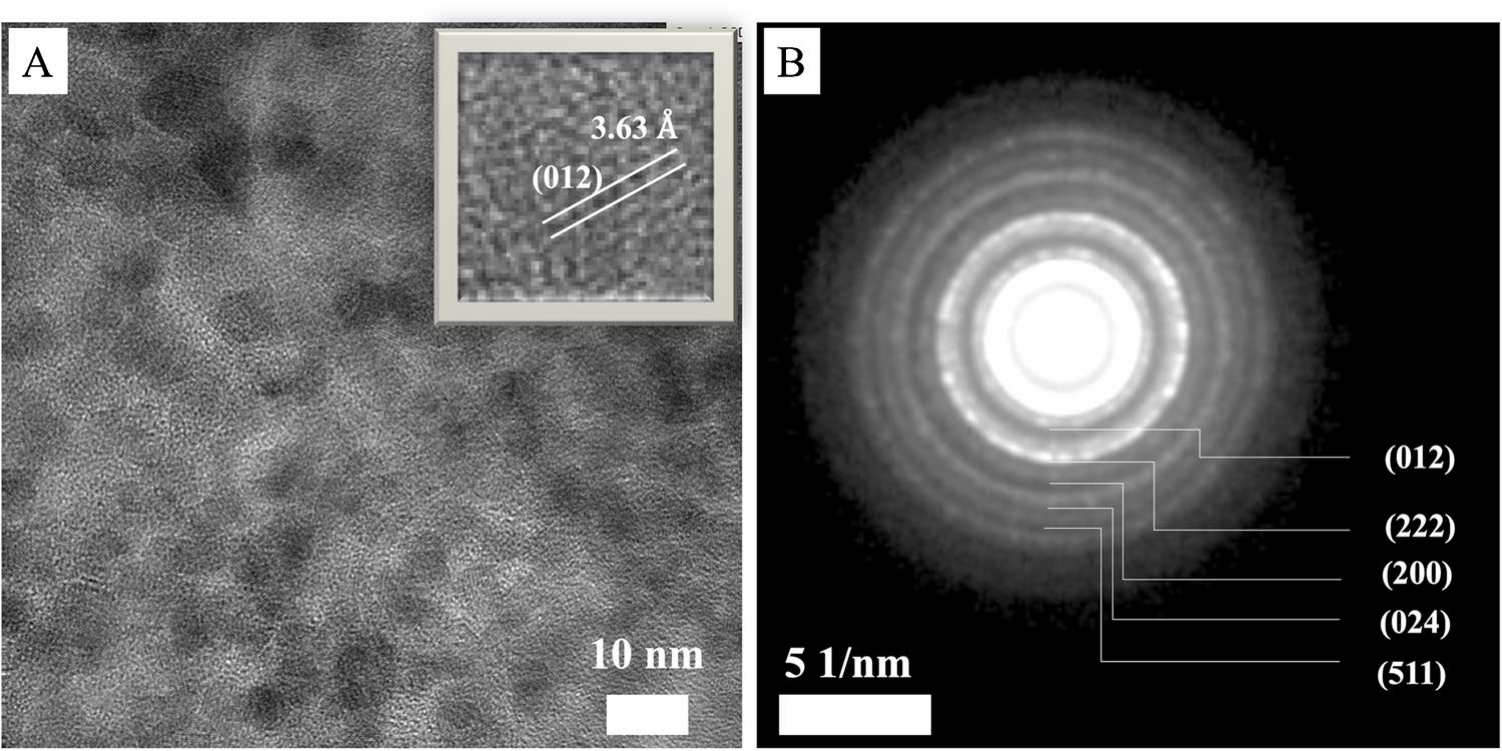
**A** HRTEM and **B** SAED images of LpAB-FeNP

UV–vis spectroscopy played a significant role in the synthesizing of FeNP, LpAB-FeNP, and its conjugation with anti-VEGF. Fig. S1 shows absorbance at 370 nm due to the metallic iron oxide core. Fig. S2A shows the absorbance at 370 nm due to a metallic iron oxide core that confirms the presence of LpAB-FeNP [46]. The decrease in the absorbance intensity after the conjugation process indicates the successful attachment of anti-VEGF to LpAB-FeNP [47, 48]. In this study, the morphology of LpAB-FeNP and LpAB-FeNP/anti-VEGF was observed by scanning electron microscopy (SEM), as depicted in Fig. S2B and Fig. S2C. As shown in Fig. 1, the LpAB-FeNP nanoparticles exhibited a globular shape. However, after the conjugation of anti-VEGF, the morphology tended to form agglomerations and appeared as cloudy clusters. This change in morphology provides evidence of the attachment of anti-VEGF to LpAB-FeNP.

The HRTEM characterization reveals that the core size of FeNPs is in 5.5 ± 1.5 nm, where ~ 5 nm spherical ones were in the most abundant form. Selected area diffraction (SAED) patterns reveal that the FeNPs had (012), (222), (200), (024), and (511) *hkl* indices. The *hkl* (012) and (024) belong to α-Fe_2_O_3_, the *hkl* (200) belongs to FeO (wustite), and (222) and (511) *hkl* indices belong to Fe_3_O_4_ (magnetite) iron oxide crystal structures [49]. Besides, the d-spacing measured on an individual FeNP (the inset in Fig. 1A) shows that the FeNP had α-Fe_2_O_3_ form.

Zeta potential and size distribution of LpAB-FeNP and LpAB-FeNP/anti-VEGF were characterized by DLS. The average size of the LpAB-FeNP was measured to be approximately 33.9 nm, with negative zeta potential − 10.1 ± 2.11. DLS recognizes hydrodynamic size while TEM only recognizes size of the metallic core, so the size difference between the two techniques is expected. After the conjugation, the size of LpAB-FeNP/anti-VEGF increased to 74.4 nm, with a zeta potential of − 13.2 ± 2.01. The increase in hydrodynamic particle size and the difference in zeta potential confirmed the successful covalent modification.

**Fig. 2:**
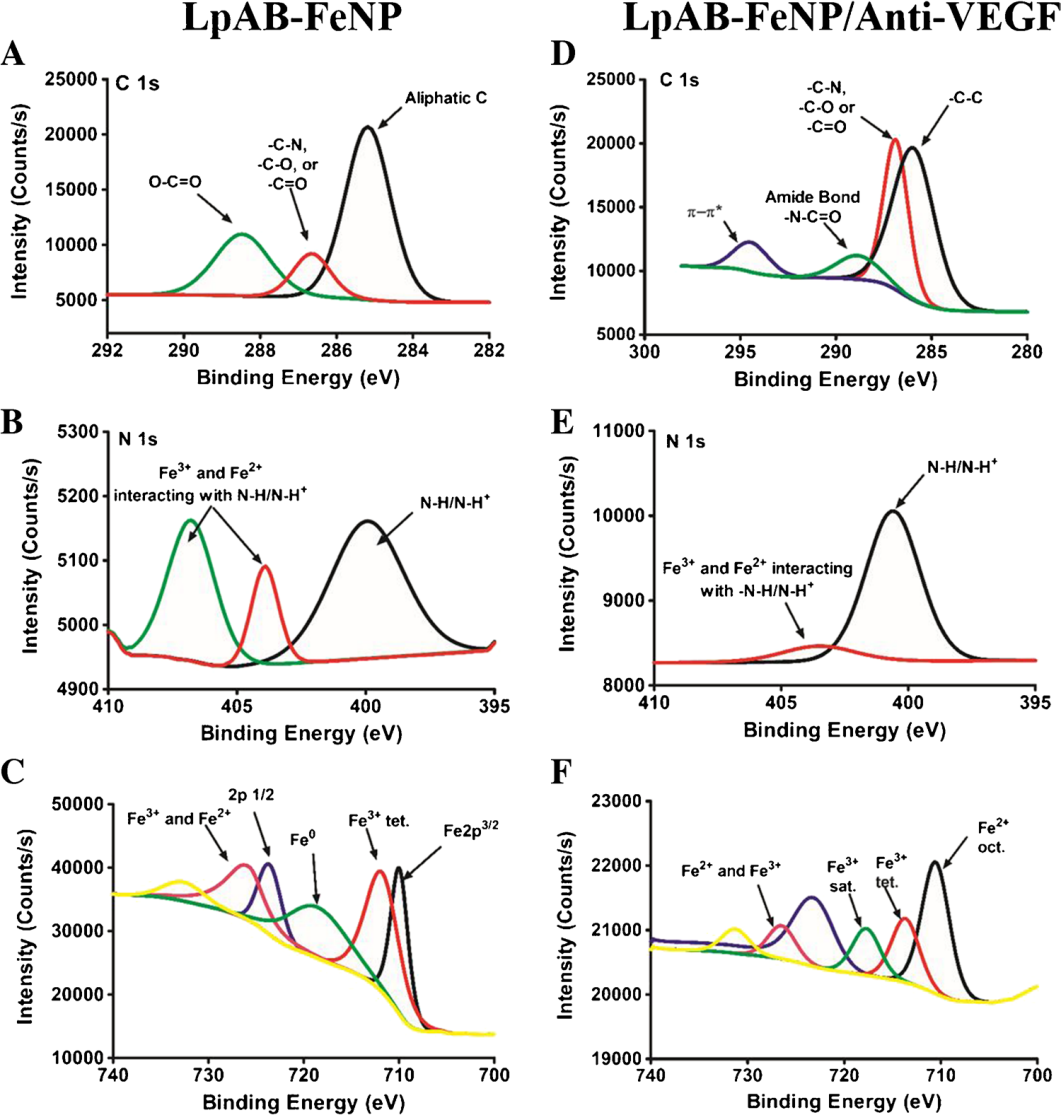
XPS spectrum of LpAB-FeNP. **A** C 1 s LpAB-FeNP. **B** N 1 s LpAB-FeNP. **C** Fe LpAB-FeNP. **D** C 1 s LpAB-FeNP/anti-VEGF. **E** N 1 s LpAB-FeNP/anti-VEGF. **F** Fe LpAB-FeNP/anti-VEGF

X-ray photoelectron spectroscopy (XPS) measurements are highly accurate and provide precise details about the chemical composition of the target species. In this study, XPS was used to thoroughly examine the binding between the conjugated molecules. Therefore, LpAB-FeNP samples and LpAB-FeNP/anti-VEGF conjugates were studied using XPS (Fig. 2). Figure 2A and Fig. 2D show Fig. 2C 1 s photoelectron spectra of LpAB-FeNP and LpAB-FeNP/anti-VEGF, respectively. Figure 2A shows three main peaks of C 1 s in LpAB-FeNP spectrum. The components centered at 285.18 eV for C, 286.68 eV for C-N/C-O or C = O, and 288.38 eV for O-C = O resulted from the presence of carboxylic acid of the sugar ligand. The C 1 s spectrum for the LpAB-FeNP/anti-VEGF surface was deconvoluted into four peaks (Fig. 2D). The peaks were observed at 286.18, 286.98, 288.88, and 294.78 eV belong to C–C bond [50], C-N/C-O or C = O bonds [45], amide groups (N–C = O) [51], and π-π* interaction [52].

Figure 2B shows the N 1 s spectrum, which exhibited three peaks corresponding to N–H/N–H + [53], Fe^2+^, and Fe^3+^ interacting N–H/N–H + at 399.98, 403.98, and 406.88 eV, respectively [54]. The N 1 s deconvolution of LpAB-FeNP/anti-VEGF (Fig. 2E) gave two distinct groups of peaks. The peaks at 400.68 and 403.78 eV correspond to N–H, N–H + , N–O [55], and amide groups (N–C = O) [56], respectively, which support the covalent linkage between LpAB-FeNP and anti-VEGF. In Fig. 2C and Fig. 2E, the characteristic peak of Fe 2p3/2 is expected at 710.2 eV, which is indicative of the core level spectra of Fe_3_O_4_ nanoparticles [57]. As illustrated, the peaks in the Fe 2p spectrum are situated at 726.68 eV for LpAB-FeNP and 710.88 eV for bivalent iron (Fe^2+^) in LpAB-FeNP/anti-VEGF. Furthermore, the peaks at 712.48, 714.08, 718.38, and 726.98 eV can be attributed to trivalent iron (Fe^3+^) [58]. The component at 724.08 eV corresponds to Fe 2p_1/2_. According to the literature, the characteristic peak of zerovalent Fe (Fe^0^) is expected at 719.9 eV [56]. As shown in Fig. 2F, a characteristic peak at 719.98 eV for Fe^0^ is observed. Combining all the XPS results, it can be concluded that the anti-VEGF has been successfully conjugated onto the surface of LpAB-FeNP.

### Analytical characterization of VEGF LpAB-FeNP/anti-VEGF

**Fig. 3:**
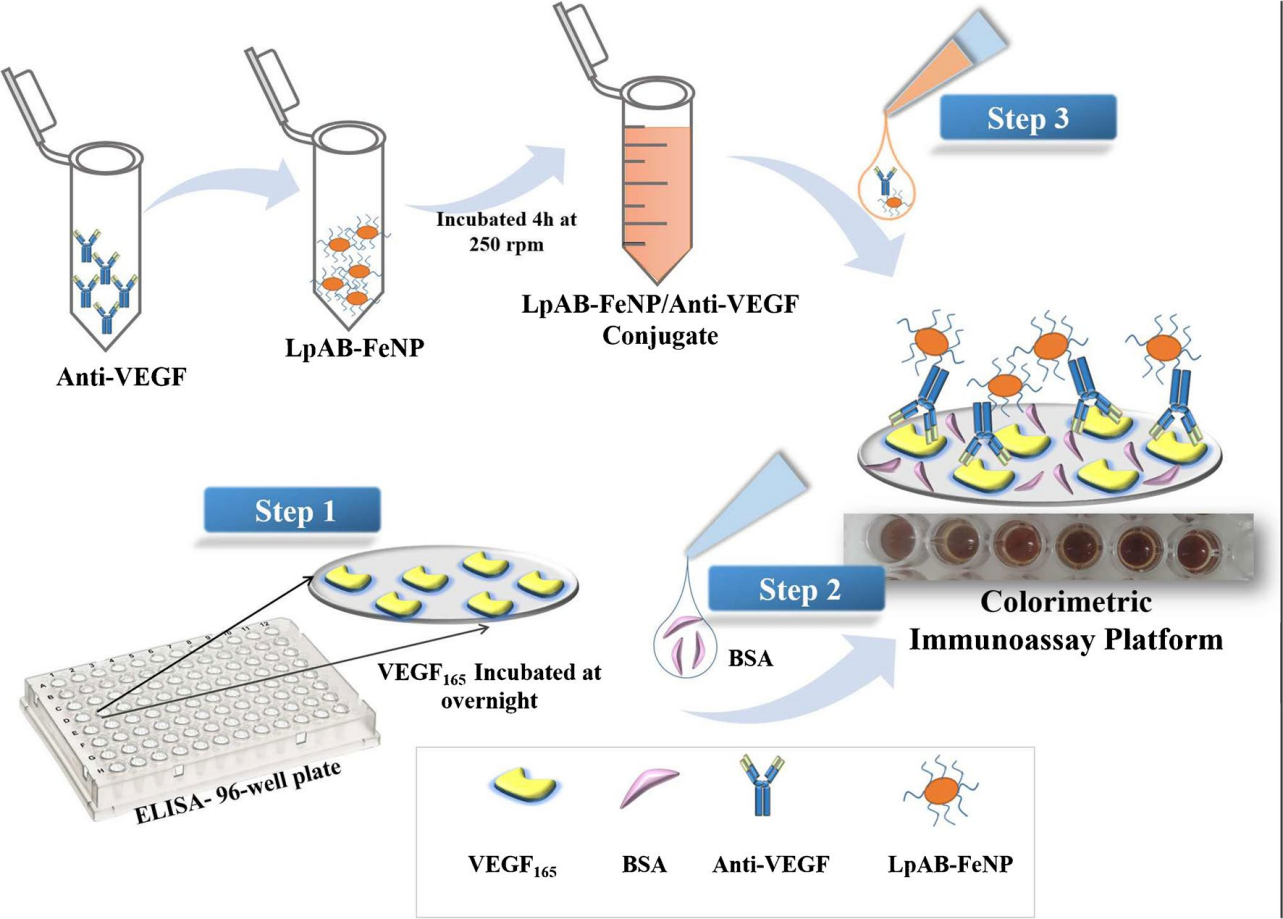
Schematic demonstration of colorimetric immunoassay preparation platform

Enzyme-mimicking nanomaterials have emerged as potential substitutes for natural proteins [59]. Similarly, an antibody-based immunoassay was developed for C-reactive protein (CRP) [45] where a metal organic framework (MOF) labeled with anti-CRP was used as a probe. In the presence of CRP, the labeled anti-CRP forms a connection via affinity, and a fluorescence signal is generated depending on the target. In this study, a colorimetric analysis of immunoassay targeting different levels of VEGF_165_ was performed using colorimetric detection in PBS buffer (pH 7.4). Here, the LpAB-FeNP/anti-VEGF conjugate was employed as the detection antibody, which was captured by VEGF_165_ coated onto a 96-well plate. LpAB-FeNP served as the source of the colorimetric signal. The schematic presentation of the proposed immunoassay is given in Fig. 3.

**Fig. 4:**
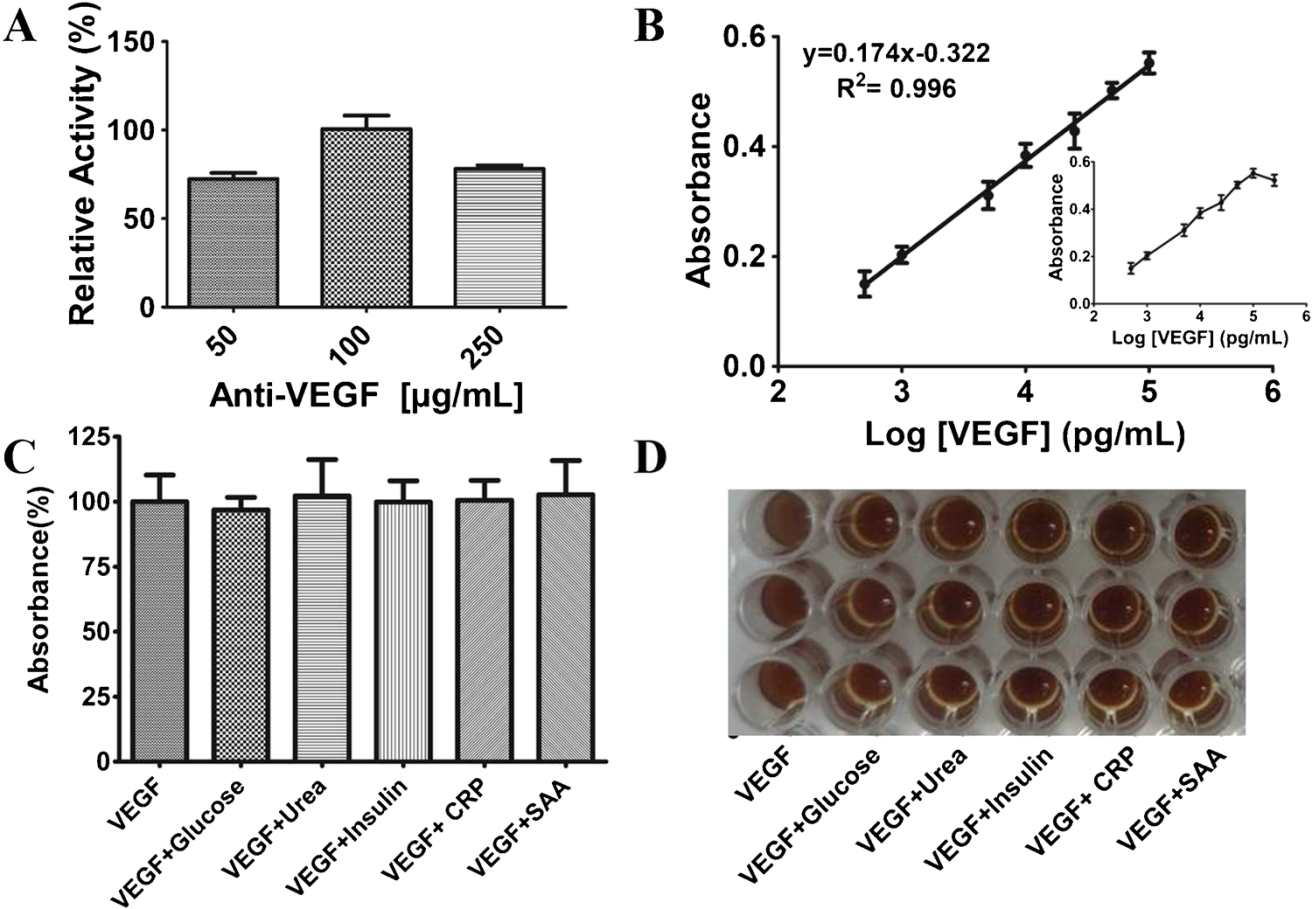
**A** Optimization of anti-VEGF concentration. **B** Calibration curve of log VEGF_165_ (pg/mL) constructed by linear fitting. **C** The result of the proposed FeNP-based ELISA assay for VEGF_165_ detection over some potentially interfering substances: VEGF_165_, 10 ng/mL; glucose, 10 mM; urea, 0.1 mg/mL; insulin, 10 μlU/mL; CRP, 10 ng/mL; SAA, 10 ng/mL. **D** Representative example of interference results obtained in the ELISA plate. Error bars show SD of at least three measurements

The investigated assay parameters for the detection were the concentration of BSA, VEGF_165_ incubation time, and the concentration of anti-VEGF. The BSA concentration and incubation time for VEGF_165_ were determined based on previous studies [45, 60]. The calibration curve of the proposed VEGF_165_ determination assay exhibited a wide linear range between 0.5 and 100 ng/mL with the best fit equation of *y* = 0.174*x* − 0.322. Absorbance values of VEGF_165_ Log (pg/mL) at different concentrations are shown in the Supplementary Materials file (Table S1). Additionally, the correlation coefficient (*R*^2^) was calculated as 0.996, indicating a reliable relationship, as shown in Fig. 4B. Figure 4D presents an image depicting the potential impact of interferences on the proposed immunoassay platform.

The limit of detection (LOD) is a crucial parameter for assessing the analytical performance of a method with a certain level of confidence [61]. In the LpAB-FeNP/anti-VEGF immunoassay, the LOD was determined using 10 measurements at the lowest concentration (0.5 ng/mL) on the calibration curve. LOD was calculated using the formula 3SD/*m*, where SD represents the standard deviation of 10 measurements performed at the lowest VEGF_165_ concentration and *m* represents the slope of the VEGF_165_ calibration curve. The experimental determination of LOD in this study was 0.29 ng/mL.

Table 1 provides an overview of the comparison of some VEGF sensors described in the literature. Based on signal transducer types, immunoassays can be classified as electrochemical, colorimetric, or optical immunoassays. Among these, colorimetric immunoassays for disease biomarker detection have drawn much interest because of their ease of use and high level of effectiveness [62].

**Table 1:** Comparison of VEGF determination studies summarized in literature

Target	Detection method	Nanomaterial	Linear detection for VEGF	LOD	Ref
VEGF	Electrochemical	RGO/Au NPs	2–20,000 ng/mL	6 fg/mL	[63]
VEGF	Fluorescence	GO/Aptamer	0.32–5.0 nM	0.32 nM	[64]
VEGF	Fluorescence	Quantum dot microspheres	25–1600 pg/mL	-	[65]
VEGF	Electrochemical	DNA aptamer	5 pM	50 pM–0.15 nM	[66]
VEGF	Electrochemical	GO/MB-AuNPs-aptamer-Fc sensing	2–500 pg/mL	0.1 pg/mL	[67]
VEGF	Electrochemical	AuNA@NC	0.01–10 ng/mL	6.77 pg/mL	[68]
VEGF_165_	Electrochemical	Fe_3_O_4_/Fe_2_O_3_@Au	0.01–10 pg/mL	0.01 pg/mL	[69]
VEGFR1	Electrochemical	Au/3-MPA	10–70 pg/mL	-	[70]
VEGF	Electrochemical	GO-ssDNA	0.05–100 ng/mL	50 pg/mL	[71]
VEGF	Electrochemical	Cd(II)@LP and Cu(II)@LP	0.01–7000 pg mL	0.005 pg/ mL	[72]
VEGF	Chemiluminescence	CdTe QD/H_2_O_2_	2–35,000 pg/mL	0.5 pg/mL	[73]
VEGF_165_	Colorimetric	G-quadruplex DNAzymes/Cat	24.00 pM–11.25 nM	1.70 pM	[74]
VEGF_165_	Colorimetric	LpAB-FeNP	0.5–100 ng/mL	0.29 ng/mL	This study

*RGO/Au NPS* reduced graphene oxide/Au nanoparticles, *GO* G-quadruplex aptamer, *GO/MB-AuNPs-aptamer-Fc* graphene oxide/methylene blue-AuNP-ferrocene-labeled aptamer, *AuNA@NC* Au nanoarchitecture (Au NA) embedded with nanochitosan, *Fe*_*3*_*O*_*4*_*/Fe*_*2*_*O*_*3*_*@Au* magnetic iron-gold nanoparticles, *VEGFR1* vascular endothelial growth factor receptor-1, *Au/3-MPA* gold electrode/3-mercaptopropionic acid, *GO-ssDNA* graphene oxide/ssDNA, *Cd(II)@LP* cadmium-loaded liposome, *Cu(II)@LP* cupper-loaded liposome, *Cat* catalase

In the current literature, the tests are mostly performed using electrochemical and colorimetric methods. They frequently employ aptamer/antibody combinations, offering high specificity and selectivity. Furthermore, the repeatability, stability, and affordability of the test system can be improved by using antibodies and/or aptamers [75]. In our case, the results show that the prepared LpAB-FeNP/anti-VEGF has many advantages over the others, including a better LOD and a broader linear detection range. The repeatability of the immunoassay played a key role in our VEGF_165_ measurement platform. We investigated the repeatability through conducting eight measurements in the presence of 10 ng/mL VEGF_165_ protein. The coefficient of variation (CV) of the platform was calculated as 0.152%.

The potential interference effects of glucose, urea, insulin, CRP, and SAA (common biological components found in blood) on the immunoassay response for detecting VEGF_165_ were examined. The reference levels for the interferences are 0.35–5.0 mM for glucose [76], 0.10–0.16 mg/mL for urea [77], 2–20 mIU/mL for insulin [78], lower than 0.3 to 1.0 mg/mL in healthy individuals for CRP [79], and 0.02–0.05 mg/mL for SAA under normal conditions [80] according to literature. LpAB-FeNP/anti-VEGF/VEGF_165_ was prepared, and the effect of each substance in the immunoassay response was evaluated using the colorimetric method in the presence of 10 ng/mL VEGF_165_ (Fig. 4C, D). The selectivity was calculated to be 96.84% for VEGF_165_ + glucose, 102.22% for VEGF_165_ + urea, 99.86% for VEGF_165_ + insulin, 100.54% for VEGF_165_ + CRP, and 102.67% VEGF_165_ + SAA as seen in Fig. 4C. The impact of the potential interferences was found to be lower than 5%, indicating that the immunoassay exhibited excellent selectivity. Therefore, glucose, urea, insulin, CRP, and SAA did not interfere with the LpAB-FeNP/anti-VEGF immunoassay platform.

The proposed immunosensor platform was employed to determine VEGF_165_ in the artificial blood sample. A known amount of VEGF_165_ (10 ng/mL) was added to artificial serum and determined the recovery % with LpAB-FeNP/anti-VEGF. The recovery % of VEGF_165_ detection was calculated as 95.16%, and the relative standard deviation (RSS) was calculated as 5.08. Confirming the system’s validity, the obtained result falls within the acceptable recovery range of 95–105% [81].

## Conclusion

In this study, we successfully designed a colorimetric measurement system for the detection of VEGF_165_. The developed LpAB-FeNP/anti-VEGF immunoassay platform offers several advantages, including short analysis time, high accuracy, high specificity, reduced sample volume, and low cost. Besides, the wide linear range and low detection limit, this developed simple ELISA approach reduces the utilization of extended assay time and consumption of chemicals and antibodies. Therefore, the developed platform can be an alternative method in the detection of VEGF, which can be expanded to the detection of different analytes that are critically important in diagnostics.

### Supplementary Information

Below is the link to the electronic supplementary material.

Supplementary file1 (DOC 240 KB)

## Data Availability

Data will be made available on request.
