# Supplementary material for: A colorimetric immunoassay for the detection of human vascular endothelial growth factor 165 (VEGF165) based on anti-VEGF-iron oxide nanoparticle conjugation
Source: Mikrochim Acta. 2024 Feb 14;191(3):133. doi: 10.1007/s00604-024-06228-0 (PMC10867064; doi:10.1007/s00604-024-06228-0)
Supplement: Supplementary file 1 — Supplementary file1 (DOC 240 KB) [file 604_2024_6228_MOESM1_ESM.doc]

**Electronic Supplementary Material**

**A colorimetric immunoassay for the detection of human vascular endothelial growth factor 165 (VEGF_165_) based on anti-VEGF-iron oxide nanoparticle conjugation**

Hülya Kuduğ Ceylan^1†^, Fatma Öztürk Kırbay^2^, İdris Yazgan^3^ and Murat Elibol^4^

*^1^ Department of Basic Pharmaceutical Sciences, Faculty of Pharmacy, Tokat Gaziosmanpaşa University, 60250 Tokat, Turkey.*

*^2^ Ege University Faculty of Science Biochemistry Department, 35100 Bornova, Izmir, Turkey.*

*^3^ Kastamonu University Faculty of Science and Art Department of Biology, Center for Biosensors and Material Science, 37100 Kastamonu, Turkey.*

*^4^ Ege University, Bioengineering Department, 35100 Bornova, Izmir, Turkey*

*^†^ Hülya Kuduğ Ceylan contributed this study at Ege University, Bioengineering Department, 35100 Bornova, Izmir, Turkey*

**Correspondence authors:**

Hülya Kuduğ Ceylan (hulya.kudug@gop.edu.tr)

Fatma Öztürk Kırbay (ffatma.ozturkk@gmail.com)


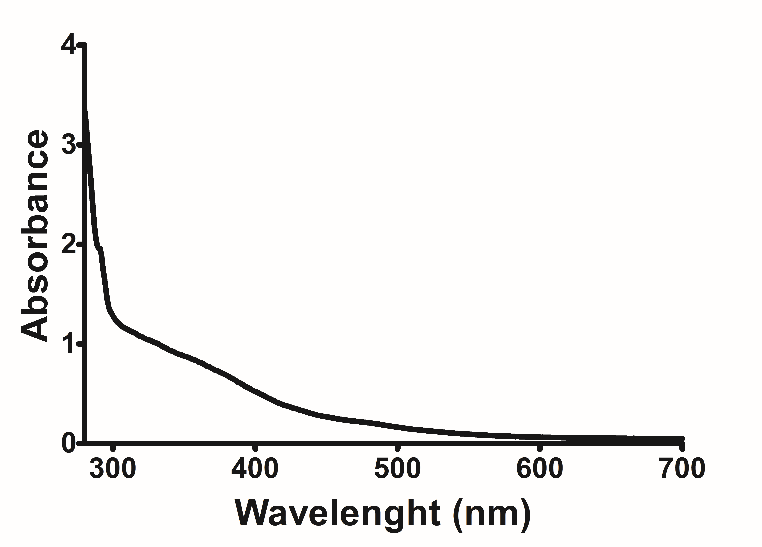


**Fig. S1** UV-Vis absorption spectra of FeNP

**
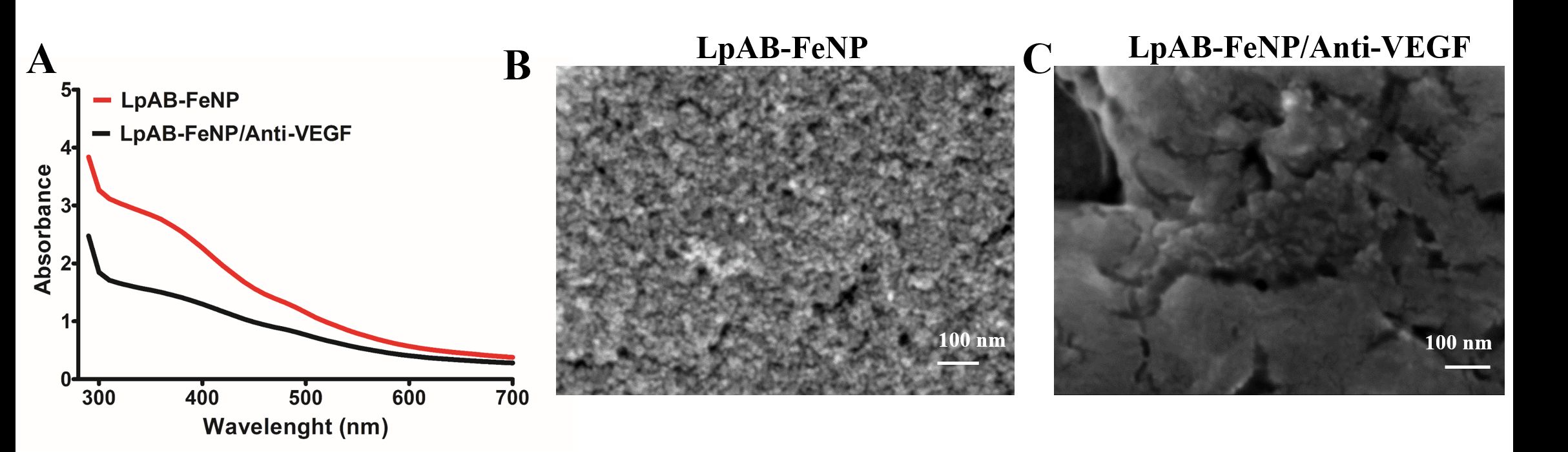
**

**Fig S2.** A) UV-Vis absorption spectra of LpAB-FeNP and LpAB-FeNP/Anti-VEGF, SEM images of B) LpAB-FeNP and C) LpAB-FeNP/Anti-VEGF

**Table S1**. Absorbance values of VEGF_165_ at different concentrations Log (pg/mL).

| **VEGF_165_**  **Concentration (ng/mL)** | **VEGF_165_**  **Concentration (pg/mL)** | **VEGF_165_**  **Concentration**  **Log (pg/mL)** | | **Absorbance***  **(n= 3)**  **± SD** | |
| --- | --- | --- | --- | --- | --- |
| 0.5 | 500 | | 2.699 | | 0.151±0.023 |
| 1.0 | 1000 | | 3.000 | | 0.203±0.015 |
| 5.0 | 5000 | | 3.699 | | 0.311±0.025 |
| 10 | 10000 | | 4.000 | | 0.384±0.021 |
| 25 | 25000 | | 4.398 | | 0.428±0.032 |
| 50 | 50000 | | 4.699 | | 0.502±0.013 |
| 100 | 100000 | | 5.000 | | 0.552±0.019 |
| 250 | 250000 | | 5.398 | | 0.522±0.024 |

* (*n*= 3) means three times of measurements. ± standard deviation (SD)
